# A realist synthesis of controlled studies to determine the effectiveness of interventions to prevent genital cutting of girls

**DOI:** 10.1179/2046905513Y.0000000086

**Published:** 2013-11

**Authors:** Rigmor C Berg, Eva M Denison

**Affiliations:** Norwegian Knowledge Centre for Health Sciences, Oslo, Norway

**Keywords:** Female genital mutilation/cutting, Female circumcision, Prevention, Realist synthesis, Systematic review

## Abstract

**Background:** Female genital mutilation/cutting (FGM/C) is a traditional practice which involves the partial or total removal or other injury to the female genital organs for non-medical reasons. Although current trends indicate that the practice is becoming less prevalent, as many as 30 million girls may still be at risk of FGM/C. Given the associated risks and violation of the human rights of girls and women, the practice is discouraged through preventative interventions.

**Aims:** To systematically review the effectiveness of anti-FGM/C interventions, applying a realist perspective.

**Methods:** The realist synthesis approach addressed context-mechanisms-outcomes (CMO) underlying intervention in an attempt to explain interventions’ success or lack thereof. The process involved exposed the usefulness of strategies in different contexts.

**Results:** Eight effectiveness studies and 27 context studies were included. All of the effectiveness studies employed a controlled, before-and-after study design. They formed five broad categories of intervention: training, formal classroom education, media communication, outreach and advocacy, and informal adult education. The CMO configurations showed that the proposed engine behind changing behaviours regarding FGM/C appeared largely to be dissemination of information. Some interventions’ acceptability and success rested on the incorporation of FGM/C abandonment efforts within a larger set of related issues. However, greater success of the interventions could have materialised with systematic pre-planning involving thorough community analyses and securing religious leaders’ commitment to halting FGM/C.

**Conclusion:** The results of this analysis point to conditions that facilitate the success of FGM/C abandonment programmes in different contexts and can be used in future prevention efforts to reduce the risk of girls being subjected to FGM/C.

## Introduction

Female genital mutilation/cutting (FGM/C) is a traditional practice, most prevalent in Africa and the Middle East, which involves the partial or total removal or other injury to the female genital organs for non-medical reasons.^1^ FGM/C comes in many forms, but four general types are described: clitoridectomy (type I), excision (type II), infibulation (type III), and other (type IV), with infibulation being the most invasive.^1^

Although current trends indicate that the practice is becoming less prevalent, as many as 30 million girls under the age of 15 may still be at risk of FGM/C.^2^ In countries where more than 70% of women aged 15–49 years live with FGM/C (e.g. Eritrea, Ethiopia, Mali and Somalia), fewer daughters than mothers have been subjected to it, and far from all women with FGM/C support continuation of the practice. For example, only 31% of women in Ethiopia believe that FGM/C should continue. In some countries such as Eritrea and Ethiopia, however, the number of girls subjected to FGM/C is greater than the number of women who support it, probably indicating that it is decided in a wider context.^2^

There are many negative health sequelae of FGM/C.^3^ Immediate complications include severe pain, bleeding and infection.^1^ Long-term consequences are recurrent bladder and urinary tract infections, infertility,^1^ attenuation of sexual functioning,^4^ and an increased risk of childbirth complications.^5^ Given the increased risk of harm and its violation of human rights,^6^ and consistent with international condemnation of FGM/C, the practice is discouraged through preventative interventions.

In a previous systematic review, the authors examined the effectiveness of interventions to reduce or prevent FGM/C of girls.^7^ In the studies included, there were few significant differences between the intervention and comparison groups. However, the meta-analysis for prevalence of FGM/C in girls aged ≤10 years showed a decrease in FGM/C among daughters of intervention participants. This and other findings indicated that the interventions had some positive effects.

Unfortunately, the effectiveness review did not assess the degree to which the interventions were appropriate responses to the populations’ needs with respect to FGM/C, including the degree to which factors that contribute to the perpetuation of the practice were taken into account in the interventions. Furthermore, the studies had different designs and presentation schemes and were implemented by researchers and organizations with different religio-political backgrounds within hugely varying cultural contexts. Although knowing the effectiveness of programmes is necessary, it is not sufficient for policymakers, researchers, activists and other stakeholders to decide which type of programme has the greatest potential for success in various situations. Thus, while our earlier programme effectiveness assessment allowed an understanding of what happened in the programmes, it could not answer *how* or *why*. To fill this gap, we conducted a realist review, which is uniquely suited to gain insights into “what is it about this programme that works for whom in what circumstances.”^8^

The realist synthesis approach addresses context-mechanisms-outcomes (CMO) configurations that underlie interventions in an attempt to explain interventions’ success and failure. This involves exposing theories of why they would work and their use in different contexts.^8^^–^10 The people behind realist review explain that “interventions offer resources which trigger choice mechanisms (M) which are taken up selectively according to the characteristics and circumstances of subjects (C), resulting in a varied pattern of impact (O).”^9^ Generative mechanisms are the engine behind behaviour (what the programme offers that may persuade participants to change). The context is important because the action of mechanisms to some extent depends on the realities of the context in which they are used. Thus, the realist approach aims to generate hypotheses of how efficacy of an intervention varies depending on the particular configuration of its constituent mechanisms and contexts.9,^10^

Applying a realist perspective, this article systematically reviews the effectiveness of anti-FGM/C interventions within a framework which allows examination of conditions and factors that facilitate or hamper the success of interventions. The review is an abridged and revised communication of a technical report.^11^

## Methods

As seen in the technical report,^11^ this systematic review followed general criteria from systematic review guidelines,12,^13^ applied standards for realist synthesis,^8^^–^10 and met the PRISMA statement criteria with regard to processing and reporting results.^14^ Methods were specified in advance and documented in a review protocol. Results of the realist synthesis are presented and readers are referred to the full technical report for further details.

### Inclusion and exclusion criteria

The systematic review incorporated randomized controlled trials, quasi-randomized trials, controlled before-and-after studies, and interrupted time series designs on the effectiveness of interventions. To identify context factors, we included cross-sectional quantitative studies, qualitative studies, and mixed-methods studies reporting context-relevant factors related to the continuance and discontinuance of FGM/C. All studies were required to meet the following inclusion criteria: to report original, empirical research, and consider girls and/or young women at risk of FGM/C, or other members of communities practicing FGM/C such as religious leaders or health workers. Any intervention intended to prevent or reduce the prevalence of FGM/C was eligible, including education, training and alternative rites. The comparator could be no FGM/C intervention (including a ‘wait list’ in which a group assigned to a waiting list receive an intervention after the active intervention group does) or other active FGM/C intervention. Intervention outcomes were comprehensive, including rates of FGM/C, behaviour and intentions related to FGM/C and attitudes towards and beliefs and knowledge related to the practice.

### Search strategy

Thirteen electronic databases [African Index Medicus, Anthropology Plus, British Nursing Index and Archive, The Cochrane Library (CENTRAL, CDR, DARE), EMBASE, EPOC, MEDLINE, PILOTS, POPLINE, PsychINFO, Social Services Abstracts, Sociological Abstracts, WHOLIS) up to March 2011 were searched using text words and medical subject headings to identify studies. In addition, searches in databases for grey literature and those of relevant international organizations were conducted. We performed bibliographic back-referencing and forward citation tracking through the ISI Web of Knowledge, and asked experts for suggestions in the literature.

### Study selection, appraisal, data extraction and analysis

The processes of study selection, quality appraisal and data extraction were undertaken by two reviewers, independently of each other. Disagreements were resolved by consensus. The full-text of any study which the reviewers agreed appeared to meet the inclusion criteria was retrieved. Once included, studies were grouped according to methodological focus: effectiveness studies, quantitative context studies, qualitative context studies. Methodological quality of studies was assessed using design-specific checklists. Standardized data extraction sheets were developed and used. Theory-linked behaviour change techniques^15^ were identified and used to characterize intervention components. For the realist synthesis, it was necessary to extract information concerning mechanisms that were assumed to underpin the intervention. This information was collected from the effectiveness reports and related publications by examining documents and extracting all statements which addressed mechanism issues. Behavioural and social ‘cogs and wheels’ of the intervention and administrative and related mechanisms were searched for.

**Figure 1:**
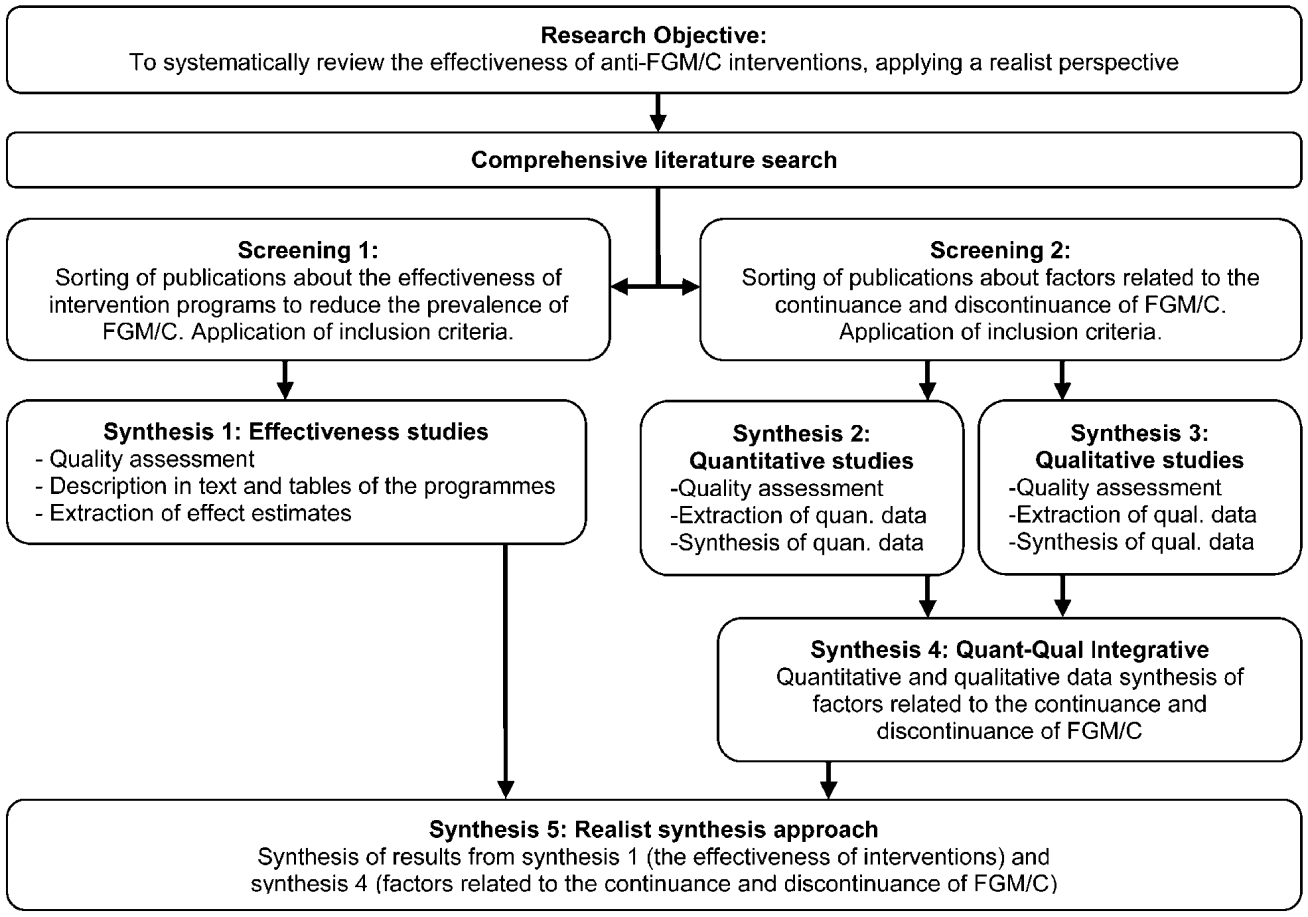
Data synthesis approach

The data analysis involved several steps. An integrative evidence approach was used in which data extraction and analyses of effectiveness data and context data were completed in separate streams first and linked in the final step (Fig. 1).

Briefly, the effects of interventions at study level by the adjusted absolute risk difference, relative risk and mean difference were estimated. Studies which were sufficiently similar were pooled using the statistical technique of meta-analysis to estimate effect (synthesis 1). A generic inverse variance approach was used for the synthesis of quantitative context studies (synthesis 2), and thematic analysis for qualitative context evidence (synthesis 3). Results from the latter were subsumed under the quantitative context results and extended them (synthesis 4). In the final step of the analysis (synthesis 5), the results of the effectiveness studies were integrated with the results from the context studies in a realist synthesis approach which aimed to address CMO configurations that underlie interventions. Recent examples of realist syntheses served as models for our approach.^16^^–^18 The effect estimates extracted from the effectiveness studies provided the outcome patterns. Regarding context, the data on context provided in the effectiveness studies and the results of the included context studies were considered. The realist synthesis involved creating matrices in which CMO data for each intervention type were juxtaposed. The interaction of these data in an interpretive, reflexive and iterative process were examined to unravel the mechanisms that were set in motion to produce the outcomes.

## Results

Electronic searches identified 6323 individual records and the manual search 472 relevant records. After removal of duplicates and exclusion of non-eligible records, the full text of 105 potentially relevant studies were screened and 35 were included. Eight of these were effectiveness studies and 27 were context studies.

### Study characteristics

All eight effectiveness studies, described in 13 publications, employed a controlled before-and-after study design and were rated for weak study quality. The studies formed five broad categories of intervention: (i) training,^19^ (ii) formal classroom education,^20^ (iii) media communication,^21^ (iv) outreach and advocacy,^22^ and (v) informal adult education.^23^^–^31 All eight studies compared an intervention group with a group that received no intervention, except one in which the control group received education.^22^ In total, the effectiveness studies involved 7042 participants (range 108–1465) at entry in seven African countries.

Twenty-seven context studies which described FGM/C in the countries in which a controlled study of an intervention to reduce the prevalence of FGM/C had been undertaken were identified. (No context studies were identified in Ethiopia). Methodological quality was assessed as high for nine studies, 12 had moderate and six had low methodological quality. The number of participants in the included context studies ranged from 67 to 15,573 (total 77,463) and included both men and women.

### Training of health personnel

One controlled study evaluated the effectiveness of training and supervising health personnel.^19^ The programme included a range of different behaviour-changing techniques and was embedded in local public health services (Table 1). It aimed to improve Malian health providers’ involvement with FGM/C, given their routine contact with people affected by FGM/C in south-east Mali where FGM/C is particularly prevalent (data from included context study).^32^

**Table 1:** Summary of the contextual factors identified, intervention, mechanisms triggered and outcomes of a training programme in Mali

Context^32^	Intervention characteristics^19^	Mechanisms	Outcomes
Mali, districts of Bamako and Bla.	Intervention (*n* = 59), 8 health clinics.	Training leads to improved knowledge, attitudes and skills.	There was no significant difference between the intervention and comparison groups in any of the outcomes at endline (knowledge, beliefs).
Health personnel: Bambara, 96% Muslim.	Comparison (*n* = 49), 6 health clinics.	Insufficient time span for training.	
94% prevalence of FGM/C (mostly types I&II), highest among ethnic groups in south. No national law/codes can be applied. Increasingly performed by health personnel, but with no formal FGM/C training. Most people support and intend to continue the practice.	Who? Health personnel (obstetricians, gynaecologists, family planning providers).		
Reasons for: tradition, religion, hygiene.	When? Duration 2 months (likely in 1997).		
Reasons against: complications, bad tradition, it prevents sexual satisfaction.	What? Group training sessions consisting of local rationale for FGM/C, its prevalence, the different types of cutting, and the health complications and treatment, plus supervision. Used 6 behaviour-change techniques, largely provision of information and instruction.		

The CMO configuration showed that the programme, implemented where FGM/C may be performed increasingly by health personnel, involved an appropriate stakeholder group with the potential to reduce the prevalence of the practice. However, the results showed that improvements were not triggered by the intervention: there was no significant difference between the intervention and comparison groups regarding any outcome. It is possible that the training did not allow adequate time for adoption of the intended attitudes or for changes in behaviour to occur. On the other hand, the intervention seemed to be a fitting response to strengthen health workers’ clinical involvement in FGM/C and prepare them to support and inform patients and communities about the desirability of discontinuing FGM/C. In Mali, the most frequently voiced reason for opposing the practice was medical complications, and thus the intervention was founded on a view already accepted in the community. However, the extent to which the programme’s “context and local rationale for FGM/C”^19^ were grounded in empirical data is not clear.

### Education of female students

In Egypt, where FGM/C is undertaken in more than nine of every ten girls, there are many groups to target in the prevention of FGM/C. Reasoning that adolescents in Egypt lack sufficient accurate knowledge of reproductive health, Mounir and colleagues^20^ studied the effect of two educational sessions on female university students’ beliefs and knowledge of reproductive health, including FGM/C (Table 2). The intervention’s ‘educational contents were based on the results of the pretest’,^20^ suggesting that the programme incorporated information on FGM/C specific to the target population. The context studies showed that over 60% of university students would favour FGM/C for their daughter and considered it a good custom.^33^^–^41 The behaviour-change technique employed (the provision of information on the consequences of FGM/C) seems sound, given that context studies showed that the most common reason for opposing the practice was fear of complications. Furthermore, in a context of near universal prevalence of FGM/C where few women recognise the possibility of adverse physical consequences of FGM/C but many believe that it can affect sexual relations, promoting sexual health in a reproductive health curriculum probably aided the programme’s success. Health education interventions have been touted as one of the most appropriate strategies for promoting young people’s sexual health,^42^ and the provision of formal education did increase students’ knowledge of the likely complications of FGM/C.

**Table 2:** Summary of the contextual factors identified, intervention, mechanisms triggered and outcomes of an education programme in Egypt

Context^33^^–^41	Intervention characteristics^20^	Mechanisms	Outcomes
Egypt (lower), urban setting in city of Alexandria.	Intervention (*n* = 354), residents of 1 hostel.	Education leads to improved knowledge and attitudes.	Reproductive health sessions for female students achieved a significant increase in mean scores for knowledge of dangers of FGM/C compared with no intervention (MD 0·75 points, 95%CI 0·63–0·87).
Female students: 47% from low social class.	Comparison (*n* = 328), residents of 1 hostel.		
99% prevalence of FGM/C in 15–19-year-olds. National law prohibiting FGM/C. Role of men important, but most think FGM/C is not important for marriage.	Who? Female students.		
Reasons for: custom and good tradition, preserve sexual morals/reduce women’s sexual desires, hygiene, religion.	When? Duration 2 hrs, likely in 2001.		
Reasons against: physical complications/harm, sexual problems, no value/benefit, religion.	What? 2×1-hr sessions on reproductive health, consisting of presentations, group discussion, role play, use of educational aids. Used 1 behaviour-change technique (provide information on consequences of behaviour to the individual).		

### Communication programme

The communication intervention was implemented in a Nigerian state with about 37% prevalence of FGM/C in communities with little or no education who considered it an essential traditional practice (Table 3). According to the intervention researchers, factors underpinning FGM/C were cultural: uncut women were considered unmarriageable, unclean, and potentially promiscuous.^21^ These results appeared to be derived from a pre-intervention study and largely mirrored the results of our included context studies.^43^^–^56

**Table 3:** Summary of the contextual factors identified, intervention, mechanisms triggered and outcomes of a communication programme in Nigeria

Context^43^^–^56	Intervention characteristics^21^	Mechanisms	Outcomes
Nigeria, south-east (Enugu state). Community members: most low education, 58% Protestant.	Intervention (*n* = 484), 3 areas in Enugu state.	Convention theory:	Communication programme for community members achieved a significant increase in the proportion of:
25% prevalence of FGM/C (37% in south-east). No national law against FGM/C. About 20% intended to cut daughter. Considered ‘compulsory’ in south-east. Support for FGM/C strongest among older, cut, less educated women.	Comparison (*n* = 473), 3 areas in Ebonyi state.	- Programme leads to increased awareness, which leads to self-examination of beliefs and values, which triggers ways of thinking and value orientations	- Women who encouraged someone not to perform FGM/C on their daughter (RR 2·68)
Reasons for: tradition, control female sexual desire/prevent promiscuity, religion, avoid pregnancy/delivery issues.	Who? Male and female community members.	- Programme leads to dialogue and group/ social interactions and advocacy, which in turn improves self-efficacy and perceived social support.	- Women with no intention of performing FGM/C on their daughter (RR 1·13)
Reasons against: physical complications/harm, unnecessary/bad tradition.	When? Duration ∼12 months (2003–2004).	- High degree of programne exposure improved FGM/C-related attitudes, but 36·6% not exposed	- Men who did not believe there were benefits from FGM/C (RR 1·17)
	What? Multimedia communication (e.g. newspapers, radio call-in shows), development of action plans to improve women’s situation, community meetings. Used 5 behaviour-change techniques, largely provision of information and goal-setting.	- Programme exposure through mass media and community activities affected change more than exposure through either one alone.	- Men who believed most community members favoured discontinuation of FGM/C (RR 1·76).

Babalola and colleagues explained that the programme aimed to ‘changing FGC-related attitudes and promoting the intention not to perform FGC.’ Several behaviour-change techniques were used and the postulated change theory for the intervention was the convention theory (Table 3). According to this theory, conventions lie behind the stability of institutions and tradition, and can also explain rapid change. Specific to FGM/C, Mackie^57^ posits that the practice emerged as a strategy to secure marriage and the theory predicts that change in FGM/C results from co-ordinated abandonment in intramarrying groups so as to preserve a marriage market for girls not cut.

In this Nigerian context^43^^–^56 of an intervention that did not require literacy, it appears that a strategy of facilitating group interactions centred around FGM/C in the community and promoting advocacy with peers improved not only attitude towards the practice but advocacy also. There was evidence of a shift in perspective regarding FGM/C through the provision of knowledge and the actions of some which spread to others through social networks: value orientation, advocacy and perceived social support improved. Rather than outright condemnation, it seems the programme promoted careful reflection on what FGM/C meant in the target culture and why it was perpetuated. Consistent with convention theory, education about FGM/C, public discussions and declarations of opposition to FGM/C all contributed to developing a critical mass of individuals who changed their beliefs about FGM/C. It seems that cultural factors underpinning the continuation of FGM/C were embedded in the communication intervention, which is likely to have contributed to the success of the programme. There was a sound fit between the programme’s theory of change and core components. In this context, with convention theory a driver of change, dosage of programme messages seemed important as outcome data documented a clear advantage of exposure to a combination of activities and mass media. Presumably, progress could have been greater had more community members been exposed to the communication programme.

### Outreach and advocacy

Two controlled studies evaluated the effectiveness of an outreach and advocacy intervention^22^ (both reported in one publication). The intervention was implemented amongst Somalis in refugee camps in Kenya and among the Afar people of Ethiopia, two Muslim communities without laws prohibiting FGM/C and in which the practice is almost universal (data from^22^ and one included context study^58^). Using several behaviour-change techniques and change theories (Table 4), the intervention linked FGM/C to a wider health agenda and made concerted efforts to engage religious leaders. This decision seems to have been derived from pre-intervention research to understand the context of the practice, revealing a strong link between FGM/C and Islam among Somalis and Ethiopians.

**Table 4:** Summary of the contextual factors identified, intervention, mechanisms triggered and outcomes of outreach and advocacy in Ethiopia and Kenya

Context^58^	Intervention characteristics^22^	Mechanisms	Outcomes/conclusions
Kenya, refugee camp for Somalis. Community members: adult, Muslim, little education.	Intervention (*n* = 720), Ifo refugee camp.	- Education leads to increased awareness	Outreach and advocacy for community members achieved significant increase in the proportion of people in *comparison* group who believed that FGM/C compromised the human rights of women (RR 0·77).
100% prevalence of FGM/C (mainly type III). Kenyan law against FGM/C in 2001.	Comparison (*n* = 720), Hagadera refugee camp.	- Training and education trigger advocacy	
Reasons for: ensuring marriageability, religion, protection of virginity, tradition.	Who? Male and female community members in camps.	- IEC activities (Information/education/communication) affect intentions	
	When? Duration 18 months (2001–2002).	- IEC activities lead to individuals’ improved knowledge and attitudes, which leads to groups’ increased mutual understanding and agreement, which translates into collective action, which in turn shapes social norms.	
	What? Community-level information and education outreach plus advocacy educational events, community meetings, theatre group performances, video sessions, mass media activities and support of advocacy. Used 4 behaviour-change techniques, largely provision of information, instruction and prompts for identification as role model.	- Programme changes were possible and staff were trusted	
		- FGM/C addressed as part of a larger set of reproductive health issues.	
		- Community objected to intervention	
		- Planned work with religious leaders did not occur; leaders gave mixed messages	
		- Insufficient programme exposure; some messages not recalled	
		- Somalis objected to law against FGM/C	
		- Likely distrust by Muslim program recipients towards Christian program implementers.	
Ethiopia, near Awash town (north-east). Community members: Afar, Muslim, little education.	Intervention (*n* = 407), 6 villages.	- Education leads to increased awareness	Outreach and advocacy for community members achieved a significant increase in the proportion of community members who
91% prevalence of FGM/C (mainly type III). National law against FGM/C in 2004.	Comparison (*n* = 419), 6 villages.	- Training and education trigger advocacy	- had no intention to perform FGM/C (RR 2·62)
Strong link between FGM/C and Islam.	Who? Male and female community members.	- IEC activities affect intentions	- believed that FGM/C compromised the human rights of women (RR 2·21)
	When? Duration 15 months (2001–2002).	- IEC activities lead to individuals’ improved knowledge and attitudes, which leads to groups’ increased mutual understanding and agreement, which translates into collective action, which in turn shapes social norms.	- knew of harmful consequences of FGM/C (RR 1·37).
	What? Community-level information and education outreach plus advocacy educational events, community meetings, theatre group performances, video sessions, mass media activities, and support of advocacy. Used 4 behaviour-change techniques, largely provision of information, instruction and prompts for identification as role model.	- Programme changes were possible and staff were trusted – FGM/C was addressed as part of a larger set of reproductive health issues	
		- Intervention succeeded in mobilizing religious leaders	
		- Community objected to intervention	
		- Insufficient exposure to intervention.	

Our CMO assessment showed that, in an Ethiopian context, when the intervention succeeded in exposing participants to anti-FGM/C messages and mobilizing religious leaders, it triggered an improvement in knowledge of harmful consequences of FGM/C, belief that it compromised the human rights of women, and intentions not to perform FGM/C in the future. Conversely, in a similar context of need involving Somali refugees, the intervention failed to generate significant change. One critical factor that probably impeded the programme’s success among Somalis was the mixed engagement of religious leaders. In the comparison camp, several religious leaders continued their previous advocacy against FGM/C, while in the intervention camp religious leaders declined to do so. Secondly, it is likely that the outreach and advocacy intervention did not work in the Somali context because of insufficient exposure to the programme. Thirdly, the programme was implemented by the National Council of Churches of Kenya and it is possible that efforts by a Christian group to end a practice which is closely linked with Islam antagonised the target community. There was hostility in both countries to the programme and its staff for publicly addressing the negative consequences of FGM/C, but more so in the Somalian context than in the Ethiopian one.

### Tostan education programme

Three of the eight identified interventions employed the same programme and were implemented in neighbouring countries in west Africa: Mali, Senegal and Burkina Faso.^23^^–^31 In all three, there were national laws or codes relating to FGM/C, but there was still a >70% prevalence of FGM/C in the intervention areas (Table 5). These were rural villages where the beneficiaries had had no or little education, and the dominant reasons for supporting the practice were tradition and religion (data from included context studies).32,^59^^–^61

**Table 5:** Summary of the contextual factors identified, intervention, mechanisms triggered, and outcomes of Tostan education programme in Mali, Senegal and Burkina Faso

Context32,^59^^–^61	Intervention characteristics^23^^–^31	Mechanisms	Outcomes
Mali, Kati area (south). Community members: Bambara, Muslim, no or little education.	Intervention (*n* = 132), 5 villages.	- Education leads to increased knowledge, which fosters confidence + empowerment, which affect a sense of activism	Tostan education programme for community members achieved an increase in the proportion of intervention participants opposed to FGM/C, but there was a baseline difference between the groups regarding this outcome (8% in intervention group *vs* 25% in comparison group).
94% prevalence of FGM/C (mostly types I&II). Especially ethnic groups in south. No national law/codes can be applied. Most support and intend to continue.	Comparison (*n* = 107), 4 villages.	- Education affects intentions, attitudes, and skills	
Reasons for: tradition, religion, hygiene.	Who? Male and female community members.	- Education leads to public discussions	
Reasons against: complications, bad tradition, prevents sexual satisfaction.	When? Duration 6 months (2000).	- Education increases empowerment, which affects attitudes and behavior.	
	What?: Tostan four-module education programme (hygiene, problem-solving, human rights, women’s health). Used 4 behaviour-change techniques: provision of information, goal-setting, and prompts for practice and identification as role model.	- Programme ‘grounded’ in local context	
		- Programme included both genders	
		- Separate women’s circles were important for reinforcement	
		- Human rights framework was meaningful to participants	
		- Participants received FGM/C information from sources other than the intervention.	
		- Drop-out from sessions, especially men	
		- Insufficient pre-service facilitator training	
		- Disagreements, lack of mutual expectations among organisers	
		- Implementation problems	
		- Lack of clarity how to fit into local context.	
Senegal, Kolda region (south). Community members: Pulaar and Mandingo, Muslim, no or low education.	Intervention (*n* = 949), 20 villages.	- Education leads to increased knowledge	Tostan education programme for community members achieved a significant
28% prevalence of FGM/C (94% in Kolda) (mostly type I&III). National law against FGM/C in 1999. About half approved of FGM/C	Comparison (*n* = 383), 20 villages.	- Education leads to improved attitudes and skills	- decrease in the proportion of 0–10-year-old girls who were cut (RR 0·77)
Reasons for: respect tradition, obey religious demand, for cleanliness, initiation of girls, for women to get married, men prefer cut women.	Who? Male and female community members.	- Education leads to public discussions/social interactions, which leads to public commitment	- increase in the proportion of women who knew at least two consequences of FGM/C (RR 2·92)
	When? Duration 6 months (2001).	- Education empowers people.	- increase in the proportion of men who knew at least two consequences of FGM/C (RR 3·10).
	What? Tostan four-module education program. Used 2 behaviour-change techniques: provide information on consequences of behaviour to the individual, and prompt identification as role model/position advocate.	- Participants satisfied with the programme	
		- Only those most motivated participated in everything	
		- Participants received FGM/C information from other sources, before and during the intervention	
		- Intervention villages selected met certain criteria, many willing to abandon FGM/C.	
		- Religious leader openly favored FGM/C	
		- Many who signed up did not attend	
		- Low and inconsistent participation; high drop-out	
		- Some objected to the intervention	
		- Implementation problems.	
Burkina Faso (Zoundwéogo, south-central). Community members: Mossi, 45% Muslim, no or little education.	Intervention (*n* = 1012), 23 villages.	- Education leads to increased knowledge	Tostan education programme for community members achieved a significant increase in the proportion of
72% prevalence of FGM/C (mostly types I&II). National law against FGM/C in 1996.	Comparison (*n* = 453), 23 villages.	- Education leads to improved motivation and skills	- women who regretted having had daughter cut (RR 1·26)
FGM/C most common in rural areas, among Muslim women. Support for FGM/C strongest among cut, rural, non-educated women.	Who? Male and female community members.	- Education leads to increased knowledge, engagement and triggers confidence	- women who disapproved of FGM/C (RR 1·04)
Reasons for: tradition and custom, religion, reduce sexual desire, avoid an alleged disease of genital organs.	When? Duration 8 months (2001–2002).	- Education empowers participants.	-women who knew at least two consequences of FGM/C (RR 2·92)
Reasons against: medical complications, prohibited by law, painful experience, prevents sexual pleasure.	What? Tostan four-module education program. Used 2 behaviour-change techniques (same as in Senegal).	- Delayed implementation of part of programme	- men who had no intention to perform FGM/C on daughter (RR 1·05)
		- Difficult retaining facilitators, which disrupted programme progress	- men who disapproved of FGM/C (RR 1·10)
		- Inconsistent participation and drop-out	- men who believed FGM/C was unnecessary (RR 1·06)
		- Participants didn’t distribute information	- men who knew at least two consequences of FGM/C (RR 1·47).
		- Lack of tangible incentives to motivate participants.	

The four-module adult education programme developed by Tostan, a non-profit organization, included hygiene, problem-solving, women’s health and human rights. The programme differed somewhat between the sites with regard to implementation, and the theory was referred to differently in the various reports on the programme. However, all the reports identified encouraging participants to be role models/position advocates as central techniques for changing behaviour. The change mechanisms revolved around education affecting knowledge, skills, public discourse and empowerment (Table 5).

When Tostan was replicated with the aim of increasing empowerment through education, the results were inconsistent. In Mali, the only outcome reported was that a greater proportion of intervention participants than comparison participants were opposed to FGM/C. Although there was doubt about the validity of our meta-analyses results, following the programme in Senegal and Burkina Faso, fewer women stated that they had cut their daughter and a higher proportion of participants knew of its harmful consequences. Thus, our CMO configuration results showed that, depending on the context, the Tostan programme resulted in both negligible- and small positive effects.

The degree to which Tostan conducted pre-implementation research to understand the contexts of the practice was unclear, but a lack of such might help explain the limited effect achieved. In these patriarchal settings in which FGM/C is strongly linked to tradition and religion and in which the target audience received education on hygiene, problem-solving, human rights and women’s health that might not have addressed their needs and wants, little change in FGM/C-related beliefs and behaviour ensued. Rather, women failed to participate because ‘their husbands forbade it’^26^ and male villagers expressed hostility for not being included from the start and, once included, dominated in committees. Some beneficiaries believed ‘the programme was coming to fight against the traditional culture.’^26^ None of the included texts suggested that religion was addressed in the Tostan programme, although it was a chief factor in its continuance in all communities. In Senegal, one religious leader expressed strong support for FGM/C, which might have influenced the faithful. Also, implementation challenges may have hampered the programme’s success, including low attendance and drop-out, especially among the men, and many participants failed to act as advocate or pass on the information learned. There was also insufficient training of facilitators who were uncomfortable with the module topics, difficulties with recruiting facilitators from the target communities, difficulties with retaining facilitators, delayed execution of modules, alteration of the programme, and failure to achieve initial and ongoing consensus among those responsible for the programme. On the other hand, our realist synthesis indicated benefits of Tostan being integrated in a wider health and human rights agenda, such as greater acceptance of the programme.

## Discussion

This study’s findings, although based on intervention evaluations of low methodological quality and context studies of predominantly moderate-to-high quality, indicate potentially useful knowledge of what may work for whom in what settings to arrest the practice of FGM/C. Using a realist synthesis methodology allowed us to assess not only the effectiveness of interventions but also what facilitated the (limited) success of these interventions.

The mechanisms identified in the intervention studies corresponded mainly to two behaviour-changing techniques,^15^ i.e. technique 2 (provide information on consequences of behaviour to the individual) which was found in all but one of the interventions, and technique 30 (prompt identification as role model/position advocate) which was found in six of the eight interventions. A change mechanism underpinning all interventions was that providing information about FGM/C would increase knowledge – and all but one that it would improve attitudes. Thus, it seems that the driving force for changing FGM/C-related behaviour was thought to be the dissemination of information.

On the whole, this reflects local efforts which have historically concentrated on education and advocacy.

The general implication for future programmes to reduce the prevalence of FGM/C is that gathering appropriate and sufficient data before developing a strategy to address a group’s particular needs and wishes will facilitate a positive outcome. Specifically, where there is a strong link between FGM/C and religion, programme planners must attract religious leaders’ support and commitment. It is possible that campaigns with greater focus on religious interpretation of the custom’s undesirability will be more effective than one that stresses health complications or violation of human rights. Similarly, where FGM/C is practiced widely and is a deep-seated tradition, it would be sensible to frame efforts to cease FGM/C within a larger set of related issues, such as parental desire for the health and well-being of their daughters. The involvement of skilled, community-based facilitators with background characteristics similar to those of the target population will help to ensure that the language and messages used are relevant, appropriate and make the target population relate better to a sensitive, context-bound issue such as FGM/C.

The results of this analysis point to conditions that facilitate the success of FGM/C abandonment programmes in different settings. Health professionals in countries which practice FGM/C, advocates, educators, law-makers and organizations such as the UN and WHO may benefit from incorporating this knowledge into future efforts to reduce the risk of FGM/C.
